# *In Vivo* Imaging Reveals Composite Coding for Diagonal Motion in the *Drosophila* Visual System

**DOI:** 10.1371/journal.pone.0164020

**Published:** 2016-10-03

**Authors:** Yuanlei Yue, Shanshan Ke, Wei Zhou, Jin Chang

**Affiliations:** 1 Britton Chance Center for Biomedical Photonics, Wuhan National Laboratory for Optoelectronics-Huazhong University of Science and Technology, Wuhan 430074, China; 2 MoE Key Laboratory for Biomedical Photonics, Department of Biomedical Engineering, Huazhong University of Science and Technology, Wuhan 430074, China; University of Mississippi, UNITED STATES

## Abstract

Understanding information coding is important for resolving the functions of visual neural circuits. The motion vision system is a classic model for studying information coding as it contains a concise and complete information-processing circuit. In *Drosophila*, the axon terminals of motion-detection neurons (T4 and T5) project to the lobula plate, which comprises four regions that respond to the four cardinal directions of motion. The lobula plate thus represents a topographic map on a transverse plane. This enables us to study the coding of diagonal motion by investigating its response pattern. By using *in vivo* two-photon calcium imaging, we found that the axon terminals of T4 and T5 cells in the lobula plate were activated during diagonal motion. Further experiments showed that the response to diagonal motion is distributed over the following two regions compared to the cardinal directions of motion—a diagonal motion selective response region and a non-selective response region—which overlap with the response regions of the two vector-correlated cardinal directions of motion. Interestingly, the sizes of the non-selective response regions are linearly correlated with the angle of the diagonal motion. These results revealed that the *Drosophila* visual system employs a composite coding for diagonal motion that includes both independent coding and vector decomposition coding.

## Introduction

In nature, the motion vision information is vital to living animals [1]. Previous studies have shown that while flying, flies respond to approaching threats by executing rapid visually directed banked turns, and generate the escape sequences that consisted of a series of diagonal movements around yaw and roll axes [2, 3]. Thus, the visual information processing of diagonal motion is important for flying insects and other animals to successfully evade predators and perform other essential movements [2, 4].

The presence of independent coding in directionally selective neurons in animals has been previously reported. Previous studies on ganglion cells in the mammalian retina have shown that there are only four subpopulations of neurons, each of which preferentially responds to motion in one cardinal direction [5]. In addition, Maisak et al. and Takemura et al. have found specific subpopulations of T4 and T5 cells in the optic lobes of flies, which are tuned to motion in one of the four cardinal directions [6, 7]. However, how the visual system encodes diagonal motion is still unclear.

Previous studies found that there were some neurons that preferentially respond to diagonal motions in the visual cortex of rats and cats [8–10]. Similar neurons were also observed in the fly central brain [11]. Rodents have neurons that have robust direction selectivity to diagonal motion, but have no discernible spatial patterns [10, 12, 13]. Currently, there are two possible coding mechanisms for the detection of diagonal motion: (1) independent coding by segregated neurons selectively responding to different directions [9, 10], or (2) vector coding of a neuronal population, with the result a vector sum of all involved neurons [14, 15].

The *Drosophila* visual system is a classic model that provides excellent opportunities to study the structures and organization of visual neural circuits and to explore neuronal signal-processing [16–19]. The neuroanatomical architecture of the *Drosophila* motion vision circuit has been well studied [7, 20]. The original motion signal is received by R1-R8 photoreceptor cells in the retina and is then transmitted through interneurons L1 and L2 to the lamina, where it results in distinct ON or OFF signals. Ultimately, the direction of the motion is perceived in the T4 and T5 cells in the medulla [6, 7]. Both morphological and calcium imaging data indicate that axon terminals of T4 and T5 cells project to the lobula plate and form four adjacent layers corresponding to motion in the four cardinal directions [6, 7]. This provides an independent topographic map that encodes cardinal directions and allows us to study the coding of complex motion patterns, such as those seen in diagonal motion.

In this study, we labeled motion-detection neurons, T4 and T5, with the genetic calcium probe GCamp5 and recorded the response of their axon terminals in the lobula plate to diagonal motions using *in vivo* two-photon calcium imaging in *Drosophila* [21]. Further, we investigated the coding mode for diagonal motion in the visual system.

## Materials and Methods

### Flies

Flies were maintained on a 12:12-hour light:dark cycle at 25°C with 60% humidity on Bloomington standard recipe food. Experiments were performed on 3–5-day-old female flies, with the following genotype: Uas-GCamp5G/+; Gal4-R42F06/+.

### Fly preparation

A modified method was applied to prepare samples for analysis [22, 23]. Briefly, flies were anesthetized on ice and then fixed on a custom holder with cyanoacrylate glue (Pattex, Germany). The brain of the fly was bent anteriorly by approximately 85° to expose the posterior surface. After fixing the fly on the holder, the legs and wings of the fly remained free moving. The posterior of the brain was exposed, making microscopic surgery convenient. To avoid brain vibration, we removed the M16 muscle and fixed the extended proboscis with glue. In HL-3 saline (70 mM NaCl, 5 mM KCl, 4 mM MgCl_2_, 10 mM NaHCO_3_, 115 mM sucrose, 5 mM Trehalose, 5 mM triethylsilane, 1.5 mM CaCl_2_), a small window was cut to expose the targeted neurons using sharpened #5 forceps. This approach allowed us to image the T4 and T5 cells on the transverse plane of the lobula plate.

### Visual stimulation

The motion stimulation consisted of the movement of a grating pattern. The stimulator was modified from a previous study [24]. The stimulator had a height of 65 mm and a diameter of 145 mm, and was constructed using 14 pieces of modular LED panels (SUNSCOB, China). Each panel contains 8 × 8 individual LEDs. The emission wavelength was 590 nm, the half-width of the peak was 14 nm. The display spanned 180° in azimuth and 53° in elevation while the fly was positioned at the center. The speed of the movement of the grating pattern was 1 Hz (bars moving at 26.5° per second). For local field stimulation, we used an eighth of the left 100° of the visual field in azimuth. We applied the following sequence of stimulation patterns: first, bars staying still for 15 seconds; then bars moving for 4 seconds; at the end, bars stopping and staying still for 20 seconds.

### Image acquisition and processing

We obtained images using a two-photon microscope (Zeiss LSM 780NLS, Germany) with a water immersion objective (20×, NA = 1.0). We used an excitation wavelength of 920 nm from a femtosecond pulse laser (Maitai HP from Spectra-Physics, USA). Images were analyzed using a custom-written program (Matlab, USA). Raw images were initially smoothed using a Gaussian filter (HSIZE = [10 10], SIGMA = 5). The relative fluorescence change was calculated using the formula ΔF/F = (F_stim_−F_0_) / F_0_, in which F_stim_ was obtained by averaging all images during the grating movement, and F_0_ was obtained by averaging all images taken during the two seconds before stimulation. ΔF/F images were normalized to their maximum value.

The ΔF/F values of each pixel were coded in pseudo-color images (0–255, blue to red) in Figs 1C, 1D, 2A, 3D, 4A, and 5B. A threshold (25%) was then used to determine the contour of the activated region on a topographic map, as previously reported [6]. The analysis below is all based on this threshold. For the data shown in Figs 1E, 3A, 4B, 5D and 5F, pixels were assigned to the activated regions providing ΔF/F values above the threshold. Otherwise, pixels were assigned to background. The intensity of the response for each direction is defined as the maximum ΔF/F value during the movement of the grating pattern. The sizes of the activated regions were analyzed using a two-sample t-test and the intensities of the activated regions were analyzed using the Mann-Whitney U-test.

**Fig 1:**
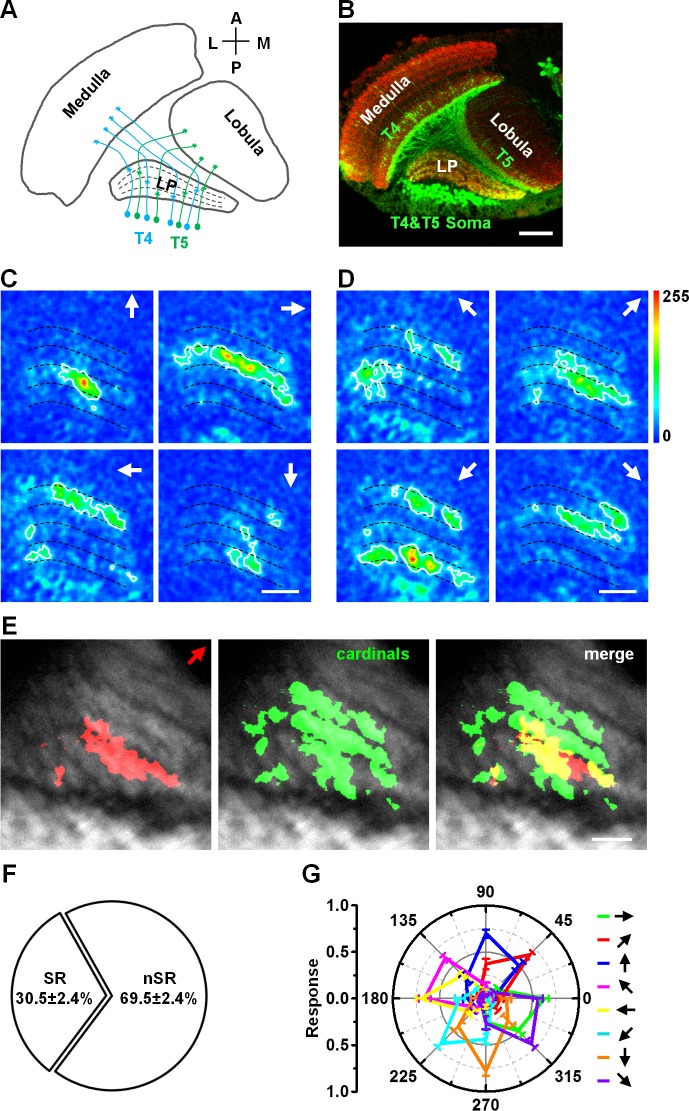
Diagonal motion activates selective response regions and non-selective response regions in the lobula plate. (A) The schematic drawing shows the projections of T4 and T5 cells and their axon terminals in the lobula plate (LP). (B) Expression pattern of Gla4-R42F06 in the lobula plate. T4 and T5 cells are labeled with GFP and the neuropil is labeled by Mouse-anti-nc82 (red). Scale bar: 20 μm. (C) Topographic maps of T4 and T5 axon terminals responding to motion in the four cardinal directions (arrows indicate the directions). (D) Topographic maps of T4 and T5 axon terminals responding to motion in the four 45° diagonal directions. (E) Diagonal motion (45° frontward to upward) activates selective response (SR) regions (red area in the right panel) and non-selective response (nSR) regions (yellow area in the right panel). These regions are compared to those responding to the four cardinal directions of motion (Green area in the middle panel). (F) The proportions of the two region types activated by diagonal motion. Statistical significance was assessed using the two-sample t-test, p < 0.001. n = 15. (G) Intensities of responses for diagonal motions and cardinal directions of motion (colors represent areas selective to the indicated directions). n = 15. Data are presented as the mean ± SEM. Statistical significance was assessed using the Mann-Whitney U-test. Scale bar: 10 μm.

**Fig 2:**
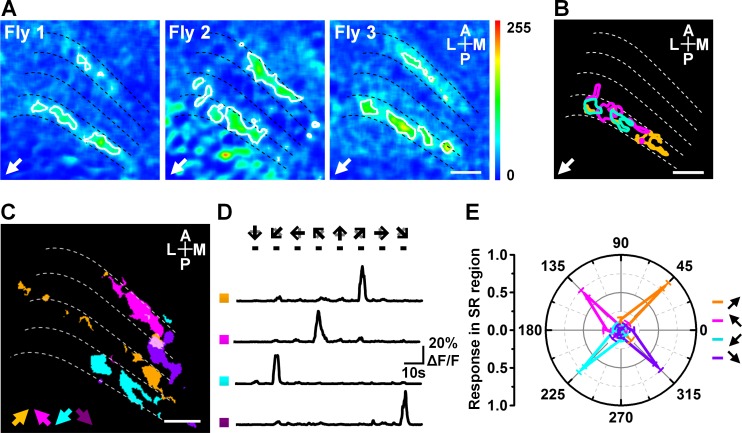
Locations and responses of the SR regions to diagonal motion. (A) Topographic maps of the response to the 45° backward to downward diagonal motion obtained from three flies. (B) Compound representation of the three SR regions in (A). The yellow, magenta, and cyan circles indicate the SR regions responding to the 45° backward to downward motion in three flies. (C) Compound representation of the SR regions responding to motion in the four 45° diagonal directions. Arrows indicate directions. (D) Calcium transients from the four regions indicated in (C). Arrows indicate the direction of grating motion. (E) Intensities of the responses to the diagonal motions in SR regions. Data are presented as the mean ± SEM. Statistical significance was assessed using the Mann-Whitney U-test. n = 15. Scale bar: 10 μm.

**Fig 3:**
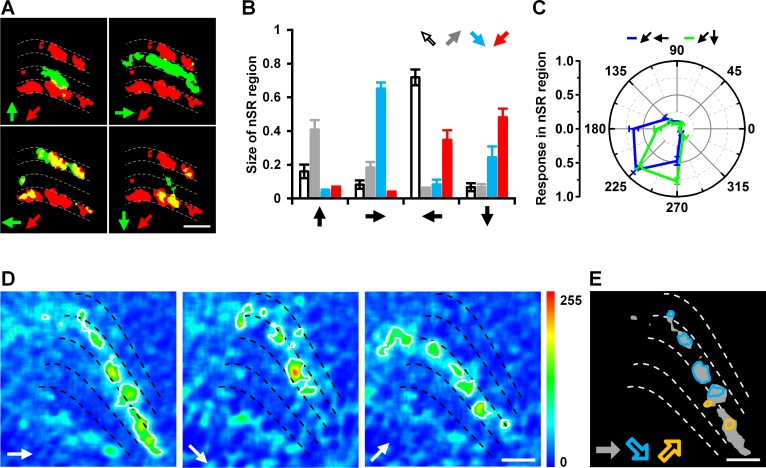
Regions activated in response to motion in the two vector-correlated cardinal directions combined to code the diagonal motion. (A) Compound display of the regions activated in response to diagonal motion (45° backward to downward) and motion in the four cardinal directions. Red and green represent regions activated by motion in the diagonal and cardinal directions, respectively; yellow represents the nSR regions that respond to both diagonal and cardinal. (B) The sizes of nSR regions activated by diagonal motion and the four cardinal directions of motion. Data are presented as the mean ± SEM. Statistical significance was assessed using the two-sample t-test. n = 15. (C) Intensities of responses in two nSR regions of 45° backward to downward diagonal motion. The blue circle represents the nSR region activated by this diagonal and backward motions, the green circle represents the nSR region activated by this diagonal and downward motions. Data are presented as the mean ± SEM. Statistical significance was assessed using the Mann-Whitney U-test, p = 0.683. n = 15. (D) Topographic maps of responses to frontward, 45° frontward to downward, and 45° frontward to upward motions. (E) The nSR regions activated by 45° frontward to downward and 45° frontward to upward are separate in a region activated by frontward motion alone. Gray areas represent the regions activated by frontward motion. Color circles represent the nSR regions responding to frontward and diagonal motions. Scale bar: 10 μm.

**Fig 4:**
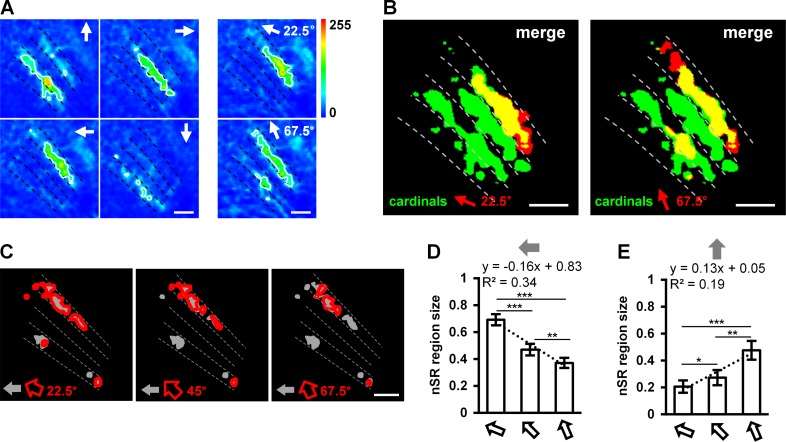
Diagonal motions are coded by the sizes of nSR regions. (A) Topographic maps of T4 and T5 axon terminals responding to motion in the four cardinal directions, and 22.5° backward to upward and 67.5° backward to upward motions (arrows indicate directions). (B) Diagonal motions of 22.5° and 67.5° backward to upward activate SR regions (red area) and nSR regions (yellow area). Regions responding to diagonal motion are compared to those responding to the four cardinal directions of motion (green area). (C) nSR regions of diagonal motion along different angles. (D) The sizes of the nSR regions responding to backward motion and the three backward to upward diagonal motions. (E) The sizes of the nSR regions activated by upward motion and the three backward to upward diagonal motions. Data are presented as the mean ± SEM. Statistical significance was assessed using the two-sample t-test, * p < 0.05, ** p < 0.01, *** p < 0.001. n = 15. Scale bar: 10 μm.

**Fig 5:**
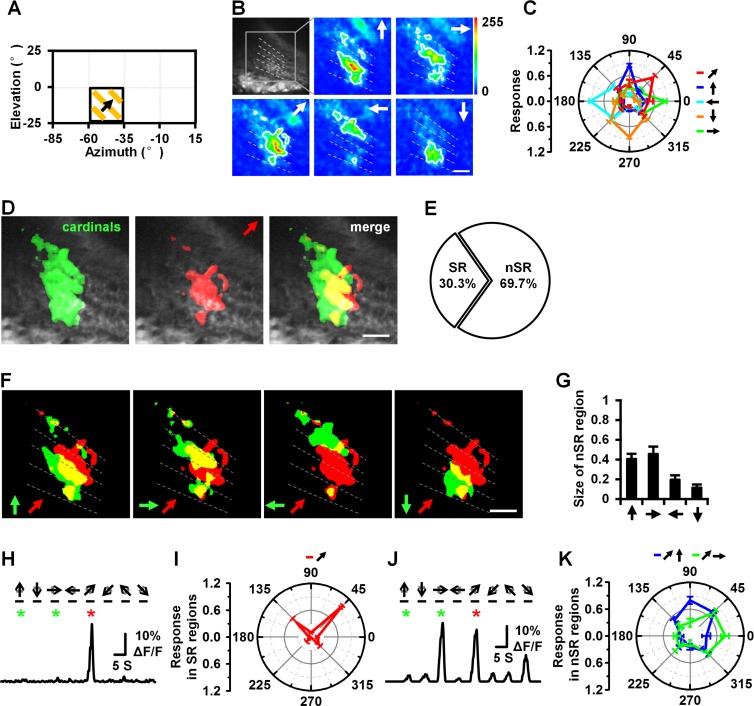
Response pattern of diagonal motion from local field stimulus. (A) Diagram of stimulation of local field (about 25° in azimuth and elevation). (B) Topographic maps of T4 and T5 axon terminals responding to motion in the four cardinal directions and 45° frontward to upward diagonal direction. (C) Intensities of responses in regions activated by 45° frontward to upward diagonal motion and cardinal directions of motion. (D) A comparison of regions responding to movement in 45° frontward to upward diagonal motion to those responding to movement in the four cardinal directions. Diagonal motion of 45° frontward to upward activate SR regions (red area in the merge image) and nSR regions (yellow area in the merge image). (E) The proportions of the SR and nSR regions for diagonal motion. (F) Compound display of the regions activated by diagonal motion (45° frontward to upward) and motion in the four cardinal directions of motion. Red and green regions represent regions activated by diagonal and cardinal directions of motion, respectively; yellow represents the nSR regions between diagonal and cardinal directions of motions. (G) The sizes of nSR regions between those activated by diagonal motion and motion in the four cardinal directions. (H), (J) Calcium transients from the SR regions (H) of 45° frontward to upward diagonal motion and nSR regions (J) of 45° frontward to upward diagonal motion and frontward motion. (I), (K) Responses in SR (I) and nSR (K) regions of 45° frontward to upward diagonal motion. The blue circle in (K) represents the nSR region activated by diagonal and upward motion, the green circles in (K) are the responses of the nSR region activated by diagonal and frontward motion. Relative fluorescence changes were normalized to the maximum response before averaging. Data are presented as the mean ± SEM. n = 6. Scale bar: 10 μm.

## Results

We investigated information coding for visual motion directions in *Drosophila*. The calcium indicator GCaMP5 was specifically expressed in T4 and T5 cells using the Gal4/UAS system [10, 18]. *In vivo* two-photon imaging technology was used to record calcium signals from the axon terminals of T4 and T5 cells in the lobula plate (Fig 1A and 1B). During the motion of the grating pattern in the four cardinal directions (upward, downward, frontward, and backward), the axon terminals of the T4 and T5 cells were activated in specific sublayers of the lobula plate (Fig 1C), forming a topographic map for the cardinal directions of motion, as previously reported [10]. To determine whether diagonal directions also have a topographic map on the lobula plate, four diagonal movements of the grating pattern (45° backward to upward, 45° frontward to upward, 45° backward to downward, and 45° frontward to downward) were applied as visual stimuli. We observed distinct activated regions in the lobula plate for different diagonal motions (Fig 1D).

To clarify the relationship between the two response patterns resulting from diagonal and cardinal directions of motion, we first compared the location of the resulting activated regions. For example, in response to the 45° frontward to upward motion (Fig 1D), the regions activated during the diagonal motion do not completely overlap with those activated during the cardinal directions of motion. These regions are divided into two groups: 1) a diagonal motion selective response (SR) region (red area in the merged image of Fig 1E), and 2) a diagonal motion non-selective response (nSR) region (yellow area in the merged image of Fig 1E) that overlaps with the response regions of the cardinal directions of motion. The same pattern of activation is also found in response to the other 45° diagonal motions. Our analysis reveals that the nSR region occupies about two-thirds of the activated region for 45° diagonal motion (Fig 1F). We also compared the intensities of the regions activated in response to diagonal and cardinal directions of motion. The results indicate that there were no significant differences in the intensities of these regions (Fig 1G). Taken together, our data indicate that two types of response regions are involved in the coding of diagonal motion in *Drosophila*. The distributions of the activated regions also imply that there is a novel response pattern for diagonal motion in the lobula plate of *Drosophila*.

We investigated the characteristics of the diagonal motion SR regions. Our results indicate that those regions activated by each diagonal motion distribute at the same layer with different locations among individual flies (Fig 2A and 2B). We also found that diagonal motion SR regions activated in response to the four 45° diagonal motions were separate from each other (Fig 2C), only responded to one diagonal motion, and were silent during motion in other directions (Fig 2D). In addition, the intensities of these regions were not significantly different (Fig 2E). These results indicate that the diagonal motion SR regions could contribute to the detection of the direction of diagonal motion. Our data also imply that diagonal motion has an independent coding mode in motion-detection neurons in flies.

In order to investigate the characteristics of the diagonal motion nSR regions, we analyzed their sizes, the intensities of their responses, and their composite features. First, we compared the sizes of nSR regions between each diagonal motion and the four cardinal directions of motion. For example, the regions activated by 45° backward to downward motion overlapped with those regions activated by backward (34.8 ± 5.7%) and downward (48.1 ± 5.2%) motions. However, these regions rarely overlapped with those activated by upward (5.6 ± 1.5%) and frontward (3.0 ± 1.0%) motions (Fig 3A). Statistical analysis of responses to the other three diagonal motions (45° backward to upward, 45° frontward to upward and 45° frontward to downward) revealed that the nSR regions activated by one diagonal motion mainly overlap with regions activated by the two vector-correlated cardinal directions of motion (Fig 3B). We then analyzed the intensities of the responses in the nSR regions and found that there were no significant differences between responses to diagonal and cardinal motions (Fig 3C). These results suggest that the coding of diagonal motions in the nSR region was closely related to that of the two vector-correlated cardinal directions. Next, to further study the regional coding of motion, we compared the nSR regions activated by one cardinal direction of motion (frontward) and two orthogonal diagonal motions (45° frontward to upward and 45° frontward to downward). The results indicate that the nSR regions responding to frontward motion and 45° frontward to upward are completely separate from the regions responding to frontward motion and 45° frontward to downward motion (Fig 3D and 3E). These data suggest that orthogonal diagonal motions are coded by distinct parts of the regions activated by motion in the same cardinal direction.

To investigate whether the above rule also applies to other diagonal directions, we performed the same analysis for other diagonal directions (by changing the angle between the backward and upward movements to 22.5° and 67.5°, respectively) (Fig 4A). Our results indicated that the coding patterns were the same for motion along those diagonal directions, as they all included SR and nSR regions (Fig 4B). However, the sizes of the nSR regions were linearly correlated with the diagonal angle (22.5°: 0.69 ± 0.04, 45°: 0.47 ± 0.04, 67.5°: 0.37 ± 0.04; y = -0.16x + 0.83, R^2^ = 0.34) (Fig 4C and 4D). We reached the same conclusion regarding the nSR regions responding to upward motion and the three diagonal motions (Fig 4E). Therefore, we suggest that the angles of the different diagonal motions are coded by the sizes of the nSR regions.

To verify the coding features of diagonal motion, we applied a local field stimulus of 25° in azimuth and elevation (Fig 5A). We recorded the response to four cardinal and four diagonal motions (45° backward to upward, 45° frontward to upward, 45° backward to downward, and 45° frontward to downward) (Fig 5B and 5C). Regions activated by diagonal motion in a local field stimulus also demonstrated an SR region and an nSR region, similar to a wide field stimulus (Fig 5D–5K). The response patterns from both wide field and local field stimuli suggest that the *Drosophila* visual system employs regional composite coding to detect diagonal motion.

## Discussion

Investigating responses to diagonal motion in the visual system is a good model for the study of information coding in neuronal circuits. In this study, we demonstrated the presence of a novel topographic map for coding diagonal motion in the motion-detection neurons of *Drosophila*. Specifically, we report that flies encode diagonal motions using regional composite coding. Thus, an SR region responds to diagonal motion, while an nSR region is involved in vector coding.

In this work, the results from calcium imaging reveal the SR regions activated by diagonal motion on the axon terminal of T4/T5 neurons. It suggests the function of T4/T5 neurons is diverse, some of them can detect cardinal motions, and the others are preferred to sense diagonal motions. This property of diversity is consistent with the previous structural study on T4 and T5 motion detection circuit. 3D reconstruction of images from series-section electron microscopy indicated that single T4 or T5 neurons receive input from multiple upstream neurons in multiple columns (T4 input from Mi1, Tm3, L5, C2 and C3; T5 input from spatially segregated Tm1, Tm2, Tm4, and Tm9), and also showed that the receptive field of individual T4 and T5 neurons covers multiple ommatidia, respectively [7, 25]. Furthermore, it is also supported by a recent functional study that shows single T4 neurons preferentially response to diagonal motion [26]. Thus, it suggests that diagonal motion could be encoded by an independent neural circuit. Although lobula plate tangential cells have been recognized as the downstream neurons of T4 and T5 [27, 28], recent studies have indicated that there are other types of neurons in the lobula plate. These neurons include lobula plate intrinsic interneurons and Hx neurons, which can receive information regarding the direction of visual motion [29, 30]. These results imply that there may be several neuronal circuits for the detection of visual motion in the lobula plate.

The overlap of the region activated by diagonal motion with the regions activated by two vector-correlated cardinal motions (Fig 3 and Fig 4) supports the population vector hypothesis for motion information coding [14, 31]. This hypothesis considers each neuron to be a vector and assumes that the direction is coded by the vector sum of the response of all neurons. Our results suggest that vector coding is involved in the detection of diagonal motion in fly.

Here, we reveal a novel pattern for the coding of diagonal motions by the axon terminals of T4 and T5 neurons in the lobula plate. We also report that the different response regions (SR and nSR regions) show differential preferences for motion in different diagonal directions. Direction and orientation are essential information for motion detection in animals. A recent report also indicated that individual T4 and T5 axon terminals display prominent orientation selectivity, and that this selectivity sharpens directional tuning via surround inhibition [29]. The co-extraction of orientation and direction information by T4 and T5 neurons may represent the first order of processing in the visual circuit. The preference and selectivity of the T4 and T5 axons may conform to a retinotopic organization, which is universal in the visual systems of insects, mammals, and primates [8, 11, 32].

Our study suggests that regional composite coding, which may combine independent coding with vector coding in neural circuits, processes diagonal directional information. It is consistent with the efficiency of the nervous system, where a small number of neurons code for motion in a wide number of directions.

## Supporting Information

S1 File Manuscript data. Data used in preparation of Figs 1F, 1G; 2E; 3B, 3C; 4D, 4E; 5C, 5E, 5G, 5I and 5K.
(XLSX)

## References

[1] Hupe JM, Rubin N. The oblique plaid effect. Vision Res. 2004;44(5):489–500. 10.1016/j.visres.2003.07.013 14680775

[2] Muijres FT, Elzinga MJ, Melis JM, Dickinson MH. Flies evade looming targets by executing rapid visually directed banked turns. Science. 2014;344(6180):172–177. 10.1126/science.1248955 24723606

[3] Gilbert C, Gronenberg W, Strausfeld NJ. Oculomotor control in calliphorid flies: head movements during activation and inhibition of neck motor neurons corroborate neuroanatomical predictions. J Comp Neurol. 1995;361(2):285–297. 10.1002/cne.903610207 8543663

[4] Ziskind AJ, Emondi AA, Kurgansky AV, Rebrik SP, Miller KD. Neurons in cat V1 show significant clustering by degree of tuning. J Neurophysiol. 2015;113(7):2555–2581. 10.1152/jn.00646.2014 25652921 PMC4416580

[5] Briggman KL, Helmstaedter M, Denk W. Wiring specificity in the direction-selectivity circuit of the retina. Nature. 2011;471(7337):183–188. 10.1038/nature09818 21390125

[6] Maisak MS, Haag J, Ammer G, Serbe E, Meier M, Leonhardt A, et al A directional tuning map of Drosophila elementary motion detectors. Nature. 2013;500(7461):212–216. 10.1038/nature12320 23925246

[7] Takemura S, Bharioke A, Lu ZY, Nern A, Vitaladevuni S, Rivlin PK, et al A visual motion detection circuit suggested by Drosophila connectomics. Nature. 2013;500(7461):175–181. 10.1038/nature12450 23925240 PMC3799980

[8] Lee KS, Huang X, Fitzpatrick D. Topology of ON and OFF inputs in visual cortex enables an invariant columnar architecture. Nature. 2016;533(7601):90–94. 10.1038/nature17941 27120162 PMC5350615

[9] Ohki K, Chung SY, Kara P, Hubener M, Bonhoeffer T, Reid RC. Highly ordered arrangement of single neurons in orientation pinwheels. Nature. 2006;442(7105):925–928. 10.1038/nature05019 16906137

[10] Ohki K, Chung S, Ch'ng YH, Kara P, Reid RC. Functional imaging with cellular resolution reveals precise micro-architecture in visual cortex. Nature. 2005;433(7026):597–603. 10.1038/nature03274 15660108

[11] Seelig JD, Jayaraman V. Feature detection and orientation tuning in the Drosophila central complex. Nature. 2013;503(7475):262–266. 10.1038/nature12601 24107996 PMC3830704

[12] Van Hooser SD, Heimel JAF, Chung S, Nelson SB, Toth LJ. Orientation selectivity without orientation maps in visual cortex of a highly visual mammal. J Neurosci. 2005;25(1):19–28. 10.1523/Jneurosci.4042-04.2005 15634763 PMC6725193

[13] Sun W, Tan Z, Mensh BD, Ji N. Thalamus provides layer 4 of primary visual cortex with orientation- and direction-tuned inputs. Nat Neurosci. 2016;19(2):308–315. 10.1038/nn.4196 26691829 PMC4731241

[14] Tanabe S. Population codes in the visual cortex. Neurosci Res. 2013;76(3):101–105. 10.1016/j.neures.2013.03.010 23542219 PMC3688279

[15] Gilbert CD, Wiesel TN. The influence of contextual stimuli on the orientation selectivity of cells in primary visual cortex of the cat. Vision Res. 1990;30(11):1689–1701. 10.1016/0042-6989(90)90153-C 2288084

[16] Clark DA, Bursztyn L, Horowitz MA, Schnitzer MJ, Clandinin TR. Defining the Computational Structure of the Motion Detector in Drosophila. Neuron. 2011;70(6):1165–1177. 10.1016/j.neuron.2011.05.023 21689602 PMC3121538

[17] Lee YJ, Jonsson HO, Nordstrom K. Spatio-temporal dynamics of impulse responses to figure motion in optic flow neurons. PLoS One. 2015;10(5):e0126265 10.1371/journal.pone.0126265 25955416 PMC4425674

[18] Gutig R, Gollisch T, Sompolinsky H, Meister M. Computing Complex Visual Features with Retinal Spike Times. PLoS One. 2013;8(1): e53063 10.1371/journal.pone.0053063 23301021 PMC3534662

[19] Borst A. Fly visual course control: behaviour, algorithms and circuits. Nat Rev Neurosci. 2014;15(9):590–599. 10.1038/Nrn3799 25116140

[20] Borst A, Haag J, Reiff DF. Fly motion vision. Annu Rev Neurosci. 2010;33:49–70. 10.1146/annurev-neuro-060909-153155 20225934

[21] Akerboom J, Chen TW, Wardill TJ, Tian L, Marvin JS, Mutlu S, et al Optimization of a GCaMP Calcium Indicator for Neural Activity Imaging. J Neurosci. 2012;32(40):13819–13840. 10.1523/Jneurosci.2601-12.2012 23035093 PMC3482105

[22] Joesch M, Plett J, Borst A, Reiff DF. Response properties of motion-sensitive visual interneurons in the lobula plate of Drosophila melanogaster. Curr Biol. 2008;18(5):368–374. 10.1016/j.cub.2008.02.022 18328703

[23] Seelig JD, Chiappe ME, Lott GK, Dutta A, Osborne JE, Reiser MB, et al Two-photon calcium imaging from head-fixed Drosophila during optomotor walking behavior. Nat Methods. 2010;7(7):535–540. 10.1038/Nmeth.1468 20526346 PMC2945246

[24] Reiser MB, Dickinson MH. A modular display system for insect behavioral neuroscience. J Neurosci Methods. 2008;167(2):127–139. 10.1016/j.jneumeth.2007.07.019 17854905

[25] Shinomiya K, Karuppudurai T, Lin TY, Lu ZY, Lee CH, Meinertzhagen IA. Candidate Neural Substrates for Off-Edge Motion Detection in Drosophila. Current Biology. 2014;24(10):1062–1070. 10.1016/j.cub.2014.03.051 24768048 PMC4031294

[26] Fisher YE, Silies M, Clandinin TR. Orientation Selectivity Sharpens Motion Detection in Drosophila. Neuron. 2015;88:1–14. 10.1016/j.neuron.2015.09.033 26456048 PMC4664581

[27] Hopp E, Borst A, Haag J. Subcellular mapping of dendritic activity in optic flow processing neurons. J Comp Physiol A. 2014;200(5):359–370. 10.1007/s00359-014-0893-3 24647929

[28] Spalthoff C, Egelhaaf M, Tinnefeld P, Kurtz R. Localized direction selective responses in the dendrites of visual interneurons of the fly. Bmc Biol. 2010;8:36 10.1186/1741-7007-8-36 20384983 PMC2876097

[29] Mauss AS, Pankova K, Arenz A, Nern A, Rubin GM, Borst A. Neural Circuit to Integrate Opposing Motions in the Visual Field. Cell. 2015;162(2):351–362. 10.1016/j.cell.2015.06.035 26186189

[30] Wasserman SM, Aptekar JW, Lu P, Nguyen J, Wang AL, Keles MF, et al Olfactory Neuromodulation of Motion Vision Circuitry in Drosophila. Curr Biol. 2015;25(4):467–472. 10.1016/j.cub.2014.12.012 25619767 PMC4331282

[31] van Hemmen JL, Schwartz AB. Population vector code: a geometric universal as actuator. Biological cybernetics. 2008;98(6):509–518. 10.1007/s00422-008-0215-3 18491163

[32] Kremkow J, Jin J, Wang Y, Alonso JM. Principles underlying sensory map topography in primary visual cortex. Nature. 2016;533(7601):52–57. 10.1038/nature17936 27120164 PMC4860131

